# The association between a priori dietary patterns and psychological disorders in military personnel

**DOI:** 10.1186/s12888-023-04650-x

**Published:** 2023-03-28

**Authors:** Karim Parastouei, Hosein Rostami, Mahla Chambari

**Affiliations:** 1 Health Research Center, Life Style Institute, Baqiyatallah University of Medical Sciences, Tehran, Iran; 2 Noncommunicable Diseases Research Center, Neyshabur University of Medical Sciences, Neyshabur, Iran

**Keywords:** Dietary pattern, Mental health, Military

## Abstract

**Background:**

Studies have identified a high prevalence of poor mental health, including depression, anxiety, and stress in military occupations. A low quality diet is one of the potential factors related to mental disorders. This study aimed to investigate the association of a priori dietary patterns, including dietary approach to stop hypertension (DASH), the Mediterranean diet (MD), dietary inflammatory index (DII), and healthy eating index-2015 (HEI-2015) with the odds of depression, anxiety, and stress in military staff.

**Methods:**

This cross-sectional study was performed on a total of 400 military staff, aged 38.67 ± 5.22 (ranging from 30 to 60) years, recruited from Iranian military centers. The dietary intake of participants and adherence to the DASH, MD, DII, and HEI-2015 was measured using a 168-item food frequency questionnaire (FFQ). Mental health was evaluated with the use of the Depression, Anxiety, and Stress Scale − 21 (DASS-21).

**Results:**

The prevalence of depression, anxiety, and stress was 64.5%, 63.2%, and 61.3%, respectively. Individuals with the highest adherence to HEI-2015, compared to those with the lowest adherence, were significantly at lower odds of anxiety (OR = 0.51, 95%CI: 0.27–0.96, p = 0.03), while higher adherence to DII was related to a 2.74-fold increased odds of anxiety (OR = 2.74, 95%CI: 1.06–7.04, p = 0.03). Compared to those with the lowest adherence (quartile 1), quartile 2 of the HEI-2015 was associated with decreased odds of stress (p = 0.04). No association was identified between dietary patterns and depression.

**Conclusion:**

Greater adherence to HEI-2015 and lower adherence to DII are associated with lower odds of anxiety in military staff.

**Supplementary Information:**

The online version contains supplementary material available at 10.1186/s12888-023-04650-x.

## Background

In recent years, there is increasing evidence accentuating the important contribution of mental health to the prevention of neuropsychiatric illnesses [1]. A number of contributing factors such as social, biological, and economic factors have been determined to be involved in the pathogenesis of depression, stress, and anxiety as the most common mental health problems all over the world [2]. However, their prevention and treatment are still a global concern [3]. The lack of effective screening tools to identify mental disorders at early stages and the low referral rates for treatment due to social labels and patients’ resistance to treatment has dramatically increased the costs of diagnosis, psychotherapy, and medication in these patients [4, 5]. However, despite these costs, very little progress has been made in reducing the burden of these diseases at the demographic level [4, 5]. Given that the available treatments are limited, identifying risk factors that threaten mental health is important to prevent these disorders. Mental health disorders are multifactorial and genetic, environmental, and lifestyle factors play a causal role in their occurrence [6].

Among environmental factors, the role of occupation in mental health has been highlighted [7]. Studies have identified high levels of mental health problems in military occupations [8]. In this regard, a study in 2017 on 1439 deployed soldiers (aged between 18 and 79) found that there are high levels of stress, anxiety, and depression among military occupations and deployed soldiers; it also showed elevated rates of panic/agoraphobia, in comparison with civilians [8]. Poor mental health in military units can cause many problems, including an increased rate of suicide, decreased performance, and even decreased immunity [9]. Research shows that stress can triple performance errors during operations [10]. Taking into account the findings of the aforementioned studies, paying attention to the mental health of employees is now one of the major concerns of military organizations because these organizations need efficient staff to carry out their missions appropriately in times of crisis [11, 12]. In different countries, to prevent mental health disorders in the military population, some programs have been implemented to control risk factors [11, 12]. An unhealthy diet is identified as one of the potential factors associated with poor mental health [13]. It has been indicated that nutrients such as omega-3 fatty acids, vitamin B12, vitamin B6, vitamin D, folate, magnesium, selenium, and zinc [14–16] and food groups such as seafood, nuts, fruits, and vegetables may reduce the odds of depression, stress, and anxiety [17]. A study on Iranian military personnel found that people with the highest adherence to healthy eating habits had 80% lower odds of depression than those with the lowest adherence [18]. While previous dietary recommendations for the prevention and treatment of mental disorders focused mainly on micronutrients or food items individually [19], nowadays a more effective and comprehensive approach for this purpose is considering dietary recommendations that cover the effect of a whole diet because people’s diets consist of a very complex set of nutrients and their interaction may have synergistic or reductive effects [20–23].

Several a priori dietary patterns, assessing the quality of the whole diet, including DASH [24], MD [25], DII [26], and HEI-2015 [27] have been suggested to be associated with mental health in the general population. But, data in this area of research is limited and inconclusive [28]. A cross-sectional study conducted in Iran on 240 university students (mean age 21.5 years; 86.7% female) showed that greater adherence to the DASH dietary pattern is associated with better mental health in Iranian university students [29]. Nevertheless, a population-based cohort study (n = 2646, mean age of 59.9 years, 49.5% women) in the Netherlands found no association between adherence to MD and DASH diets with the incident of depressive symptoms after adjustment for lifestyle factors [30]. A cross-sectional study on Iranian men and women (aged 18–55 years) identified a negative relationship between adherence to MD and odds of anxiety, depression, and psychological distress [31]. In contrast, a randomized clinical trial study on Spanish community-dwelling women aged 60 to 80 years and men aged 55 to 80 years at high risk of cardiovascular disease did not find a significant decrease in depression risk after intervention with MD [32]. In a cross-sectional study on 2047 adults from the general population (50.8% female, 50 to 69 years) in Ireland, higher DII scores were related to increased odds of depressive symptoms and anxiety in women, but not in men [33]. Noteworthy, in most cases, the diet of the military population does not conform to individuals’ food choices and preferences [34]. Therefore, available evidence from other populations is not generalizable to the military population. Given that the dietary patterns of people in different regions are widely different and there is not enough evidence regarding the relationship between diet and mental health in the military population of Iran, this study was performed to assess the relationship between DASH, MD, DII, and HEI-2015 dietary patterns with the odds of depression, stress, and anxiety in military staff of Iran.

## Methods

### Participants

The present cross-sectional study was carried out on a total of 400 Iranian male military personnel working in different military centers covered by Baqiyatallah University of Medical Science in Tehran, Iran from September 2020 to January 2022. The sample size was calculated using the Peduzzi approach [n = 10*k/P] [35], where K is the number of independent variables and P is the proportion of subjects with the outcome. Considering depression as the main outcome, with a prevalence of 16% based on the previous studies [18], and 4 independent variables (4 dietary patterns) as predictor factors, the minimum needed sample size was estimated as 250. Nevertheless, considering the availability of samples, 400 people were included in the study to maintain statistical power. Participants were randomly selected with the use of a cluster sampling approach in military centers. The inclusion criteria were: military staff having a male gender and with age ≥ 18. Subjects with any history of psychological disease or taking psychiatric drugs, anorexia nervosa or bulimia, cancers, renal and hepatic failure, colitis, adrenal diseases, cardiovascular diseases, and thyroid and parathyroid-associated diseases were excluded. Initially, data for 430 participants was available; after the exclusion of individuals who consumed anti-inflammatory, anti-obesity, and anti-diabetic drugs (n = 18), and those with missing data ( n = 1) or outrange energy intake, defined as energy intake lower than 800 or higher than 4,000 kcal/day [36] (n = 6) and subjects using multivitamin/mineral supplements (n = 5), a total of 400 subjects were included in the study. Written informed consent was taken from all subjects. The study protocol was ethically approved by the Medical Ethics Committee of the Baqiyatallah University of Medical Science, Tehran, Iran (IR.BMSU.REC.459).

### Dietary assessment

An expert nutritionist obtained dietary data through a face-to-face interview with the use of a reliable and validated semi-quantitative 168-item food frequency questionnaire (sq-FFQ), containing a list of Iranian food with standard serving sizes [37]. The FFQ includes questions about the frequency and amount of 168 food items, consumed.

during the past year. The FFQ is an appropriate tool to assess long-term dietary intakes of foods and nutrients at a group level. In this study, the FFQ was used to assess dietary patterns because of its ability to capture the usual long-term dietary patterns of participants [37]. All individuals were asked to report the frequency and amount of intake of food items per day, week, month, and year through the past year. Then, standard household measures such as cups, glasses, bowls, teaspoons, or tablespoons were used to convert the portion sizes of foods to grams/day of consumption [38]. The nutrient contents of foods were computed with the use of Nutritionist-IV software (First Databank; Hearst, San Bruno, CA, USA).

In this study, the DASH dietary pattern was calculated using the consumption of 8 food groups, including low-fat dairy products, vegetables, fruits, nuts and legumes, whole grains, red and processed meat, sodium, and sweetened beverages [39]. The amount of intake of all groups was calculated per 1000 kcal of energy intake. The scoring was based on the quintiles of consumption, so that for the consumption of low-fat dairy products, vegetables, fruits, nuts and legumes, and whole grains, the people who were in the highest quintile received a score of 5, and the people who were in the lowest quintile received a score of 1, and other subjects got a score corresponding to their consumption quintile. Also, for the consumption of sodium, red and processed meat, and sweetened beverages, the people who were in the highest quintile received a score of 1, the people who were in the lowest quintile received a score of 5, and people in other quintiles received corresponding scores. The score for each food group was added together to obtain an overall score for the DASH diet, which ranges from 8 to 40 [39].

Calculation of *HEI-2015* was done using the method of Krebs-Smith et al. [27]. The *HEI-2015* includes 13 components: (1) total fruits, (2) whole fruits, (3) greens, and (4) total vegetables, (5) total protein foods, (6) seafood and plant proteins, (7) whole grains, (8) dairy products, (9) fatty acids, (10) refined grains, 11) sodium, 12) added sugars, and 13) saturated fats. The consumption of the first six components scored between zero and 5, while the other items received a score between zero and a maximum of 10 in proportion to the amount of consumption. For components 1 to 9, the people who had the highest consumption were given the highest scores and those who had the lowest consumption were given the lowest scores, while for components 10 to 13, the subjects who used the highest amount of the associated component were given the lowest score proportionately. Finally, the score of these 13 components was summed up to compute the total score of HEI-2015, which ranges between 0 and 100.

The score of the MD was computed using the method described by Trichopoulou et al. [40], according to the intake of 8 food components, including vegetables, legumes, the ratio of monounsaturated fatty acids to saturated fatty acids (MUFA/SFA), fruits and nuts, dairy products, refined grains, red and processed meats, and fish. In this study, due to the absence of alcohol in the Iranian FFQ, the modified MD score was computed and the alcohol intake was not included in the scoring model. For dairy products, refined grains, and red and processed meats, a score of 1 was given to consumption below or equal to the median, and a score of 0 was given for the amount of consumption above the median. For the other 5 components, for the consumption equal and higher than the median, a score of 1 was given; otherwise, a score of 0 was given. The scores of all components were added to yield a total score for MD, ranging between 0 and 8.

DII was also calculated with the use of the method of Shivappa et al. [41] based on the intake of 32 food parameters, including total daily energy, protein, black and green tea, total fat, pepper, carbohydrate, iron, fiber, monounsaturated fatty acids, zinc, polyunsaturated fatty acids, omega-3 fatty acids, vitamin C, Omega-6 fatty acids, cobalamin, saturated fatty acids, trans fatty acids, cholesterol, vitamin A, selenium, thiamin, riboflavin, caffeine, pyridoxine, onion, folate, vitamin E, vitamin D, beta-carotene, magnesium, niacin, and garlic. To compute the score of DII, the mean consumption of each food parameter was standardized by subtracting the mean global consumption of food parameters from the actual subject’s consumption and dividing it by the global SD to generate z-scores. Then, z-scores were converted to proportions and centered by multiplying amounts by 2 and subtracting 1 to normalize the scoring system and avoid skewness. The centered percentile amounts for food parameters were then multiplied by the corresponding food parameter-specific inflammatory impact scores to yield the food item-specific DII score. The overall DII score was computed by summing food item-specific DII scores of all food parameters used by participants [42]. A higher DII score is representative of a more pro-inflammatory diet.

### Assessment of mental health

The prevalence of stress, depression, and anxiety was evaluated using the Depression, Anxiety, and Stress Scale-21 (DASS-21). The reliability and validity of this questionnaire have already been confirmed in the Iranian population [43]. The questionnaire contains 21 items and includes a set of three self-report subscales. Each of the 21-DASS subscales consists of 7 questions in which each question is scored from 0 (does not apply to me at all) to 3 (extremely applies to me) assessing the severity of anxiety, stress, and depression. The score of each subscale was obtained through the sum of the scores of the related questions. Since DASS-21 is the shortened form of the main scale (DASS-42), the final score of each of these subscales should be doubled [44]. A higher score indicates lower mental health. Scores ≥ 15, ≥8, and ≥ 10 were representative of having stress, anxiety, and depression, respectively [45].

### Measurement of other variables

Basic characteristics of subjects including smoking status, age, education level, marital status, recent medications, and history of diseases were obtained using a self-administered questionnaire. Physical activity was evaluated using a validated form of the International Physical Activity Questionnaire (IPAQ) and expressed as metabolic equivalent (MET)-min/week [46]. The weight and height of all individuals were measured in light clothing, without shoes, and in a standing position, with a precision of 100 g and 1 mm, respectively. Body mass index (BMI) was computed as weight (kg)/height (m^2^). Obesity was defined as BMI ≥ 30 kg/m^2^ [47].

### Statistical analyses

Scores of DII, MD, HEI-2015, and DASH were categorized into quartiles. The differences in continuous and categorical variables among quartiles of the mentioned dietary patterns were tested using the analysis of variance (ANOVA) and chi-square tests, respectively. Continuous and categorical variables were expressed as mean ± SD and frequency (percentages), respectively. Logistic regression analysis was used to determine the Odds ratio (OR) and 95% confidence interval (CI) for the relationship between the level of adherence to the dietary patterns and the prevalence of depression, anxiety and stress. The lowest quartile of each dietary pattern was considered as the reference group. Three models were fitted in logistic regression analysis. Model 1: adjusted for daily energy intake, Model 2: adjusted based on daily energy intake, physical activity, BMI, age and smoking, Model 3: Adjusted for the variables in model 2 plus marital status and education level. A value of P < 0.05 was considered as statistical significance. Statistical analysis was done using the Statistical Package for Social Science (Version 22.0; SPSS Inc., Chicago IL, USA).

## Results

A total of 400 Iranian military staff with a mean age of 38.67 ± 5.22 years (ranging from 30 to 60 years) were included in this study. The prevalence of depression, anxiety, and stress in the population was 64.5%, 63.2%, and 61.3%, respectively. The basic characteristics of the participants based on the quartiles of adherence to the HEI-2015, DII, DASH, and MD are presented in Table 1. Subjects with higher adherence to HEI-2015 significantly smoked less (P = 0.03), but no significant difference was found for other characteristics among the quartiles of HEI-2015. Moreover, no significant difference in basic characteristics was found across the quartiles of the DII and DASH diets. Individuals with higher adherence to the MD significantly had less physical activity (P = 0.009) (Table 1). Overall, the intakes of pro-inflammatory food parameters were significantly higher in the last quartile of DII, compared with the first quartile (Table S1).

**Table 1:** Basic characteristics of the participants based on the quartiles of adherence to the dietary patterns

		Quartiles of HEI-2015			Quartiles of DII			Quartiles DASH			Quartiles of MD		
	Total (n = 400)	Q1 (n = 98)	Q4 (n = 95)	P value*	Q1 (n = 100)	Q4 (n = 100)	P value*	Q1 (n = 94)	Q4 (n = 115)	P value*	Q1 (n = 106)	Q4 (n = 85)	P value*
Age (year)	5.22 ± 38.67	5.82 ± 39.26	4.82 ± 38.15	0.45	5.39 ± 38.69	3.95 ± 38.17	0.47	5.52 ± 38.97	4.59 ± 38.43	0.54	5.20 ± 38.54	4.73 ± 38.94	0.02
Height (cm)	6.19 ± 176.45	5.53 ± 177.18	6.30 ± 176.16	0.49	6.44 ± 176.32	5.41 ± 176.82	0.83	5.93 ± 176.67	6.24 ± 175.91	0.72	6.72 ± 176.45	6.14 ± 175.80	0.70
Weight (kg)	9.47 ± 78.55	10.30 ± 78.65	8.83 ± 77.25	0.45	10.23 ± 78.20	9.77 ± 79.60	0.59	7.87 ± 78.00	9.41 ± 78.13	0.09	9.04 ± 78.00	9.74 ± 77.70	0.56
BMI (kg/m^2^)	3.22 ± 25.28	3.42 ± 25.09	2.97 ± 24.94	0.46	3.17 ± 25.17	3.33 ± 25.50	0.88	2.80 ± 25.04	3.46 ± 25.33	0.12	3.09 ± 25.11	3.26 ± 25.18	0.86
Physical activity (MET-min/week)	42.96 ± 111.64	39.56 ± 113.57	44.28 ± 109.21	0.53	42.13 ± 112.78	40.99 ± 107.84	0.78	43.36 ± 108.64	41.84 ± 111.84	0.79	39.11 ± 118.59	47.51 ± 99.20	0.009
Marital status				0.49			0.98			0.42			0.91
Single	72 (18.0%)	21 (20.4%)	16 (16.8%)		18 (18.0%)	19 (19.0%)		14 (14.9%)	20 (17.4%)		21 (19.8%)	16 (18.8%)	
Married	328 (82.0%)	77 (78.6%)	79 (83.2%)		82 (82.0%)	81 (81.0%)		80 (85.1%)	95 (82.6%)		85 (80.2%)	69 (81.2%)	
Education level				0.31			0.58			0.77			0.67
Elementary	110 (27.5%)	27 (27.6%)	25 (26.3%)		23 (23.0%)	30 (30.0%)		25 (26.6%)	30 (26.1%)		30 (28.3%)	28 (32.9%)	
High school	205 (51.2%)	46 (46.9%)	50 (52.6%)		56 (56.0%)	54 (54.0%)		48 (51.1%)	57 (49.6%)		52 (49.1%)	41 (48.2%)	
University degree	85 (21.3%)	25 (25.5%)	20 (21.1%)		21 (21.0%)	16 (16.0%)		21 (22.3%)	28 (24.3%)		24 (22.6%)	16 (18.8%)	
Smoking (yes)	17 (4.3%)	8 (8.2%)	0 (0.0%)	0.03	3 (3.0%)	4 (4.0%)	0.76	6 (6.4%)	2 (1.7%)	0.10	3 (2.8%)	1 (1.2%)	0.01
Stress (yes)	245 (61.3%)	69 (70.4%)	59 (62.1%)	0.14	59 (59.0%)	69 (69.0%)	0.33	56 (59.6%)	72 (62.6%)	0.77	66 (62.3%)	54 (63.4%)	0.86
Anxiety (yes)	253 (63.2%)	70 (71.4%)	57 (60.0%)	0.24	63 (63.0%)	72 (72.0%)	0.15	66 (70.2%)	71 (61.7%)	0.41	68 (64.2%)	46 (54.1%)	0.25
Depression (yes)	258 (64.5%)	63 (64.3%)	63 (66.3%)	0.68	70 (70.0%)	70 (70.0%)	0.13	64 (68.1%)	76 (66.1%)	0.60	68 (64.2%)	56 (65.9%)	0.99
Obesity (yes)	25 (6.3%)	7 (7.1%)	6 (6.3%)	0.34	7 (7.7%)	4 (4.0%)	0.68	2 (2.1%)	8 (7.0%)	0.22	5 (4.7%)	8 (9.4%)	0.47
Hypertension (yes)	140 (35.0%)	34 (34.7%)	35 (36.8%)	0.91	40 (40.0%)	35 (35.0%)	0.64	30 (31.9%)	44 (38.3%)	0.78	41 (38.7%)	27 (31.8%)	0.73
Type 2 diabetes (yes)	16 (4.0%)	5 (5.1%)	3 (3.2%)	0.92	3 (3.0%)	2 (2.0%)	0.45	3 (3.2%)	5 (4.3%)	0.85	5 (4.7%)	3 (3.5%)	0.71
Hypercholesterolemia (yes)	11 (2.8%)	3 (3.1%)	2 (2.1%)	0.39	2 (2.0%)	5 (5.0%)	0.35	2 (2.1%)	3 (2.6%)	0.95	1 (0.9%)	3 (3.5%)	0.45

MD: Mediterranean diet; HEI-2015: Healthy Eating Index-2015; DII: Dietary inflammatory index; DASH: Dietary approaches to stopping hypertension

BMI: body mass index, MET: Metabolic equivalent of task

* P values are extracted from ANOVA analysis

.

The association of indices of diet quality with the odds of anxiety, stress, and depression is reported in Tables 2 and 3, and Table 4, respectively. After adjusting the results for potential confounders including daily energy intake, physical activity, BMI, age, marital status, level of education, and smoking, it was observed that individuals with the highest adherence to HEI-2015, compared to those with the lowest adherence, were significantly at lower odds of anxiety (OR = 0.51, 95%CI: 0.27–0.96, p = 0.03). Also, in all models, higher adherence to the DII was associated with a significant increase in the odds of anxiety; in the fully adjusted model, higher adherence to DII was related to a 2.74-fold increased odds of anxiety (OR = 2.74, 95%CI: 1.06–7.04, p = 0.03). There was no significant association between DASH and MD with anxiety (Table 2).

**Table 2:** Logistic regression analysis for the relationship between dietary patterns and odds of anxiety

		Odds of anxiety					
		Model 1^#^		Model 2*		Model 3^&^	
		p	Odds ratio (95% CI)	p	Odds ratio (95% CI)	p	Odds ratio (95% CI)
**HEI-2015**	Q1		1		1		1
	Q2	0.22	0.69 (0.38–1.25)	0.16	0.65 (0.35–1.19)	0.16	0.64 (0.35–1.18)
	Q3	0.04	0.54 (0.29–0.98)	0.02	0.50 (0.27–0.92)	0.03	0.51 (0.27–0.94)
	Q4	0.07	0.57 (0.31–1.05)	0.03	0.51 (0.27–0.95)	0.03	0.51 (0.27–0.96)
	P trend		0.05		0.02		0.02
**DII**	Q1		1		1		1
	Q2	0.88	0.95 (0.51–1.77)	0.94	0.97 (0.52–1.82)	0.92	0.96 (0.51–1.18)
	Q3	0.37	1.41 (0.65–3.06)	0.32	1.46 (0.67–3.17)	0.36	1.43 (0.65–3.13)
	Q4	0.03	2.75 (1.08–6.99)	0.03	2.80 (1.09–7.15)	0.03	2.74 (1.06–7.04)
	P trend		0.02		0.02		0.02
**DASH**	Q1		1		1		1
	Q2	0.21	0.68 (0.38–1.23)	0.22	0.69 (0.38–1.25)	0.22	0.69 (0.38–1.25)
	Q3	0.12	0.60 (0.31–1.15)	0.09	0.56 (0.29–1.09)	0.09	0.56 (0.29–1.10)
	Q4	0.20	0.67 (0.37–1.23)	0.15	0.64 (0.35–1.17)	0.15	0.64 (0.35–1.18)
	P trend		0.23		0.16		0.16
**MD**	Q1		1		1		1
	Q2	0.72	1.10 (0.63–1.92)	0.54	1.18 (0.67–2.09)	0.55	1.18 (0.67–2.09)
	Q3	0.80	1.07 (0.59–1.95)	0.92	1.02 (0.56–1.87)	0.95	1.01 (0.55–1.86)
	Q4	0.14	0.64 (0.35–1.16)	0.11	0.60 (0.32–1.12)	0.11	0.60 (0.33–1.12)
	P trend		0.17		0.11		0.11

# Model 1: adjusted for daily energy intake

* Model 2: adjusted for variables in Model 1 plus physical activity, body mass index, age and smoking

& Model 3: adjusted for variables in Model 2 plus marital status and level of education

**Table 3:** Logistic regression analysis for the relationship between dietary patterns and odds of stress

		Odds of stress					
		Model 1^#^		Model 2*		Model 3^&^	
		p	Odds ratio (95% CI)	p	Odds ratio (95% CI)	p	Odds ratio (95% CI)
**HEI-2015**	Q1		1		1		1
	Q2	0.03	0.53 (0.29–0.95)	0.03	0.53 (0.29–0.96)	0.04	0.53 (0.29–0.97)
	Q3	0.09	0.60 (0.33–1.09)	0.07	0.58 (0.31–1.06)	0.06	0.55 (0.30–1.02)
	Q4	0.35	0.74 (0.40–1.38)	0.35	0.74 (0.39–1.39)	0.35	0.74 (0.39–1.39)
	P trend		0.45		0.43		0.40
**DII**	Q1		1		1		1
	Q2	0.94	1.02 (0.55–1.88)	0.98	1.00 (0.54–1.86)	0.94	0.97 (0.52–1.82)
	Q3	0.72	1.14 (0.53–2.44)	0.80	1.10 (0.51–2.36)	0.86	1.07 (0.49–2.32)
	Q4	0.17	1.86 (0.7–4.64)	0.20	1.81 (0.72–4.54)	0.23	1.76 (0.69–4.48)
	P trend		0.17		0.20		0.22
**DASH**	Q1		1		1		1
	Q2	0.41	1.27 (0.71–2.24)	0.45	1.24 (0.69–2.21)	0.37	1.30 (0.72–2.32)
	Q3	0.78	0.91 (0.48–1.72)	0.72	0.89 (0.46–1.69)	0.77	0.90 (0.47–1.74)
	Q4	0.54	1.19 (0.66–2.12)	0.60	1.16 (0.65–2.09)	0.53	1.20 (0.66–2.17)
	P trend		0.81		0.86		0.81
**MD**	Q1		1		1		1
	Q2	0.47	0.82 (0.47–1.41)	0.53	0.83 (0.48–1.45)	0.46	0.81 (0.46–1.41)
	Q3	0.86	0.94 (0.52–1.70)	0.97	0.98 (0.54–1.78)	0.96	0.98 (0.54–1.79)
	Q4	0.99	1.00 (0.54–1.83)	0.76	1.09 (0.54–1.78)	0.79	1.08 (0.5–2.02)
	P trend		0.88		0.67		0.68

# Model 1: adjusted for daily energy intake

* Model 2: adjusted for variables in Model 1 plus physical activity, body mass index, age and smoking

& Model 3: adjusted for variables in Model 2 plus marital status and level of education

**Table 4:** Logistic regression analysis for the relationship between dietary patterns and odds of depression

		Odds of depression					
		Model 1^#^		Model 1^#^		Model 1^#^	
		p	Odds ratio (95% CI)	p	Odds ratio (95% CI)	p	Odds ratio (95% CI)
**HEI-2015**	Q1		1		1		1
	Q2	0.49	0.82 (0.46–1.45)	0.40	0.78 (0.43–1.39)	0.31	0.73 (0.40–1.32)
	Q3	0.68	1.13 (0.62–2.05)	0.85	1.05 (0.57–1.93)	0.99	1.00 (0.54–1.85)
	Q4	0.68	1.13 (0.61–2.08)	0.99	1.00 (0.53–1.86)	0.86	0.94 (0.50–1.77)
	P trend		0.47		0.75		0.89
**DII**	Q1		1		1		1
	Q2	0.11	0.60 (0.32–1.13)	0.12	0.61 (0.32–1.15)	0.20	0.65 (0.34–1.25)
	Q3	0.49	0.76 (0.34–1.66)	0.56	0.79 (0.35–1.74)	0.75	0.88 (0.39–1.97)
	Q4	0.71	1.19 (0.46–3.04)	0.68	1.21 (0.47–3.13)	0.54	1.35 (0.51–3.55)
	P trend		0.56		0.54		0.41
**DASH**	Q1		1		1		1
	Q2	0.50	0.82 (0.45–1.47)	0.52	0.82 (0.45–1.49)	0.47	0.80 (0.44–1.46)
	Q3	0.22	0.66 (0.34–1.27)	0.15	0.61 (0.31–1.19)	0.10	0.57 (0.29–1.11)
	Q4	0.76	0.91 (0.50–1.66)	0.60	0.85 (0.46–1.55)	0.58	0.84 (0.45–1.55)
	P trend		0.73		0.53		0.50
**MD**	Q1		1		1		1
	Q2	0.98	1.00 (0.58–1.74)	0.71	1.11 (0.63–1.95)	0.69	1.12 (0.63–1.98)
	Q3	0.96	1.01 (0.56–1.83)	0.96	0.98 (0.54–1.79)	0.88	0.95 (0.52–1.75)
	Q4	0.76	1.10 (0.59–2.03)	0.83	1.06 (0.57-2.00)	0.86	1.05 (0.56–1.99)
	P trend		0.77		0.94		0.99

# Model 1: adjusted for daily energy intake

* Model 2: adjusted for variables in Model 1 plus physical activity, body mass index, age and smoking

& Model 3: adjusted for variables in Model 2 plus marital status and level of education

In all models, it was shown that compared to those with the lowest adherence (quartile 1), quartile 2 of the HEI-2015 was associated with decreased odds of stress (p˂ 0.05), while people in quartile 3 of adherence to the HEI-2015 had a marginally lower odds of stress than those who had the lowest adherence to this index; however, the highest compliance (quartile 4) of the HEI-2015 was not significantly associated with the odds of stress. There was no significant relationship between DASH, MD, and DII with stress (Table 3). Moreover, no association was identified between the investigated dietary patterns and depression (Table 4).

## Discussion

Studies have identified high levels of stress, anxiety, and depression among military staff considering the intense nature of their job. This study aimed to explore the relation of DASH, MD, DII, and HEI-2015 to the odds of depression, anxiety, and stress in military staff. The findings revealed that military people who had the most compliance with HEI-2015, had 49% lower odds of anxiety; while those with high adherence to DII, had 2.74% higher odds of anxiety. Furthermore, moderate adherence to HEI-2015 was related to a significant reduction in the odds of stress.

In line with the results of this study, a prospective cohort study conducted in 2014, found no association between dietary patterns (vegetables-fruits” pattern, “snacks-drinks-milk products” pattern, and “meat-fish” pattern) and depression [48]. However, the results of an Iranian study (86.7% female) in 2020 suggested that adherence to DASH dietary pattern was correlated with better mental health among university students [49]. In addition, the meta-analysis by Nicolaou et al. found a significant inverse relationship between DASH, MD, and HEI-2010 with depression symptoms [6]. The subjects’ sex, age, occupation, energy intake, socioeconomic conditions, and the method used to assess mental health are some of the factors that might cause controversies among different studies.

In line with the current study, Burrow et al. found a link between a pro-inflammatory diet and higher anxiety severity among patients with major depressive disorders [50]. Haghighatdoost et al. also found that low DII can reduce the odds of being categorized in the top tertile of mental health disorders including anxiety in both men and women groups [51]. This can be due to the fact that many of the anti-inflammatory components of DII, such as zinc, omega-3 fatty acids, and coffee, are negatively associated with the odds of mental health disorders, and the pro-inflammatory components of this index, such as energy and carbohydrates, are associated with a higher odds of anxiety [4, 5]. However, a study conducted on US adults observed no association between a pro-inflammatory diet and anxiety [52].

Shivappa et al. suggested a 20% lower odds of developing depression in middle-aged women with the most anti-inflammatory diet compared to those with the most pro-inflammatory one [53]. Another study conducted by Salari-Moghaddam et al. suggested a significant association of DII with psychological disorders only in women [54]. Moreover, Akbaraly et al. reported that a 1SD increase in DII score can increase the possibility of depression symptoms by 66% in women, but no significant association was found in men [55]. In addition, a meta-analysis conducted in 2019, suggested that a pro-inflammatory diet estimated by a higher DII score can increase the odds of depression, especially in women [56]. It seems that the populations’ gender might affect the differences in these studies.

According to a meta-analysis by Shafiei et al., adherence to the MD was not associated with the odds of depression in cohort studies; while an inverse significant relationship was found in cross-sectional studies [57]. Their findings were in line with the results of a multicenter randomized trial which found no significant association between the MD and depression [32]. In line with the majority of the previous studies, we observed no significant relationship between MD and depression. While, the studies by Lia et al. [58] and Rienks et al. [59] found a negative association between MD and odds of depression in women, showing that gender difference may be a reason for heterogeneity in the results of the previous studies.

Gibson-Smith et al. assessed the diet quality in cases with and without depressive and anxiety disorders and revealed that depression and anxiety alone were not significantly associated with diet quality; however, they observed that the correlation between patients’ symptoms and diet quality was stronger when using MD score compared to alternative HEI [60]. Additionally, Beydoun et al. and Rahe et al. found no association between depression and diet quality measured by HEI [61, 62]. On the other hand, in a study conducted by Akbaraly et al., alternative HEI was significantly associated with the recurrence of depressive symptoms over five years; but this correlation was only reported in women but not men [63]. A study in 2020 found a significant association between HEI-2010 and depression without any sex differences [64].

Studies have suggested different mechanisms to explain the inverse association of HEI-2010 with mental health; the neural damage of stress can be reduced owing to the high amounts of PUFA, n-3 fatty acids, folate, B vitamins, and antioxidants in healthy eating patterns. Considering the association between inflammatory biomarkers and depressive symptoms, the anti-inflammatory properties of nutrients used in HEI might be effective in improving mono-amines [65, 66]. Mono-amines are a group of neurotransmitters involved in the pathogenesis of some mental diseases. The role of the monoamines noradrenaline and serotonin in diseases, such as depression is well-identified. All antidepressant medicines elevate the availability of these monoamines [67]. In contrast, our findings did not suggest any significant relationship between HEI-2015 and depression. This controversy can be explained by the subjects assessed in these studies; for example, Akbaraly et al’s study mainly consisted of office-based participants. This selective retention of the participants might have affected their results. Moreover, the subjects’ gender, age, energy intake, socioeconomic conditions, and the method used to assess mental health are some of the factors that might cause controversies among different studies.

Our findings show a significant association between higher adhesion to HEI-2015 and lower odds of developing stress. It was shown that people who were in the 2nd and 3rd quartile of compliance with the HEI-2015, had significantly lower odds of stress compared to those with the lowest compliance; but the highest compliance (fourth quartile) of HEI-2015 was not significantly related to stress. This suggests that moderate (and not high) adherence to HEI-2015 is associated with reduced odds of stress. In other words, a balance in the degree of compliance with HEI-2015 is suggested to prevent stress.

This study is among the first studies exploring the relation of diet quality to mental health in military staff. In the present study, food intake data were collected by a food frequency consumption of the recent year, which provides a long-term estimate of food intake and is less prone to error due to daily changes in food intake. Taking into account a remarkable number of confounding variables was the strength of this study; nevertheless, it should be noted that the results could be affected by other measured or unmeasured confounding factors. Moreover, the validity and reliability of the questionnaires have been confirmed. Nevertheless, some limitations should also be discussed; Assessing the mediatory effects of hormones such as cortisol and serotonin could help in better understanding the mechanism by which diet quality affects mental health. Future studies are suggested to assess the levels of hormones affecting mental health besides dietary patterns. Moreover, the cross-sectional design of this study makes it prone to bias and causality cannot be inferred. As a result, future prospective cohort studies are required. Another limitation is that this study was performed on military men, and the same results might not be expandable to women. Future studies are suggested to assess the relation of dietary patterns to the mental health of women working in military sectors. In addition, because of the participant’s job nature, data extracted from FFQ may not be representative of the general population’s dietary intake.

## Conclusion

In conclusion, higher adherence to HEI-2015 and lower adherence to DII are associated with reduced odds of anxiety in military staff. Furthermore, moderate adherence to HEI-2015 may be associated with reduced odds of stress. According to these findings, teaching healthy nutrition to military personnel in military organizations to learn and apply the HEI-2015 guidelines and reduce the consumption of foods with a high inflammatory index can be a healthy and effective nutritional approach to prevent and reduce the prevalence of mental disorders, especially anxiety and stress, in this population.

## Electronic supplementary material

Below is the link to the electronic supplementary material.

Supplementary Material 1 Supplemental Table 1. Food intakes (macronutrients and micronutrients) of participants in first and last quartiles of dietary inflammatory index (DII)

## Data Availability

The data used and analyzed during the current study are available from the corresponding author on reasonable request.

## List of abbreviations

DASH: Dietary approach to stop hypertension
MD: Mediterranean diet
DII: dietary inflammatory index
HEI-2015: healthy eating index-2015
FFQ: food frequency questionnaire
DASS-21: Depression, Anxiety and Stress Scale − 21
sq-FFQ: semi-quantitative 168-item food frequency questionnaire
MUFA: monounsaturated fatty acids
SFA: saturated fatty acids
IPAQ: International Physical Activity Questionnaire
MET: metabolic equivalent of tasks
ANOVA: analysis of variance
OR: Odds ratio
CI: confidence interval
BMI: body mass index

## References

[1] Hamilton JA, Halbreich U (1993). Special aspects of neuropsychiatric illness in women: with a focus on depression. Annu Rev Med.

[2] Cole MG, Dendukuri N (2003). Risk factors for depression among elderly community subjects: a systematic review and meta-analysis. Am J Psychiatry.

[3] Patel V, Chisholm D, Dua T, Laxminarayan R, Medina-Mora M. Global Burden of Mental, Neurological, and Substance Use Disorders: An Analysis from the Global Burden of Disease Study 2010–Mental, Neurological, and Substance Use Disorders: Disease Control Priorities, (Volume 4). 2016.

[4] Jorm AF, Reavley NJ (2012). Changes in psychological distress in australian adults between 1995 and 2011. Australian & New Zealand Journal of Psychiatry.

[5] Callegari ET, Reavley N, Gorelik A, Garland SM, Wark JD, Team S-DS (2017). Serum 25-hydroxyvitamin D and mental health in young australian women: results from the Safe-D study. J Affect Disord.

[6] Nicolaou M, Colpo M, Vermeulen E, Elstgeest LE, Cabout M, Gibson-Smith D et al. Association of a priori dietary patterns with depressive symptoms: a harmonised meta-analysis of observational studies.Psychological medicine. 2019:1–12. 10.1017/S0033291719001958 PMC7477372 31409435

[7] Pflanz SE, Ogle AD (2006). Job stress, depression, work performance, and perceptions of supervisors in military personnel. Mil Med.

[8] Trautmann S, Goodwin L, Höfler M, Jacobi F, Strehle J, Zimmermann P (2017). Prevalence and severity of mental disorders in military personnel: a standardised comparison with civilians. Epidemiol psychiatric Sci.

[9] Panjehband M, Shokraei M (2008). Evaluation of depression among soldiers in one resting place of Islamic Republic of Iran Air Force. EBNESINA.

[10] Pietrzak E, Pullman S, Cotea C, Nasveld P (2012). Effects of deployment on mental health in modern military forces: a review of longitudinal studies. J Military Veterans Health.

[11] Kobau R, Seligman ME, Peterson C, Diener E, Zack MM, Chapman D (2011). Mental health promotion in public health: perspectives and strategies from positive psychology. Am J Public Health.

[12] Teo L, Crawford C, Yehuda R, Jaghab D, Bingham JJ, Gallon MD (2017). Whole dietary patterns to optimize cognitive function for military mission-readiness: a systematic review and recommendations for the field. Nutr Rev.

[13] Marx W, Lane M, Hockey M, Aslam H, Berk M, Walder K (2021). Diet and depression: exploring the biological mechanisms of action. Mol Psychiatry.

[14] Almeida OP, Ford AH, Flicker L (2015). Systematic review and meta-analysis of randomized placebo-controlled trials of folate and vitamin B12 for depression. Int Psychogeriatr.

[15] Mocking R, Harmsen I, Assies J, Koeter M, Ruhé H, Schene A (2016). Meta-analysis and meta-regression of omega-3 polyunsaturated fatty acid supplementation for major depressive disorder. Translational psychiatry.

[16] Schefft C, Kilarski LL, Bschor T, Koehler S (2017). Efficacy of adding nutritional supplements in unipolar depression: a systematic review and meta-analysis. Eur Neuropsychopharmacol.

[17] Sánchez-Villegas A, Henríquez-Sánchez P, Ruiz-Canela M, Lahortiga F, Molero P, Toledo E (2015). A longitudinal analysis of diet quality scores and the risk of incident depression in the SUN Project. BMC Med.

[18] Rahmani J, Milajerdi A, Dorosty-Motlagh A (2018). Association of the Alternative Healthy Eating Index (AHEI-2010) with depression, stress and anxiety among iranian military personnel. BMJ military health.

[19] Ziemba JB, Matlaga BR (2015). Guideline of guidelines: kidney stones. BJU Int.

[20] Alizadeh S, Djafarian K, Alizadeh M, Shab-Bidar S. The relation of healthy and western dietary patterns to the risk of endometrial and ovarian cancers: a systematic review and meta-analysis. International Journal for Vitamin and Nutrition Research; 2019. 10.1024/0300-9831/a000514 30806608

[21] Alizadeh S, Esmaillzadeh A (2017). Meta-analysis on dietary patterns and pancreatic Cancer risk: Methodological Limitations. Nutrients.

[22] Alizadeh S, Shab-Bidar S (2019). Letter to the editor on ‘dietary patterns and endometrial cancer: a meta-analysis’. Eur J Cancer Prev.

[23] Alizadeh S, Shab-Bidar S, Mohtavinejad N, Djafarian K (2017). A posteriori dietary patterns and risk of pancreatic and renal cancers: a systematic review and meta-analysis. Nutr Food Sci.

[24] Sacks FM, Moore TJ, Appel LJ, Obarzanek E, Cutler JA, Vollmer WM (1999). A dietary approach to prevent hypertension: a review of the Dietary Approaches to stop hypertension (DASH) study. Clin Cardiol.

[25] Willett WC, Sacks F, Trichopoulou A, Drescher G, Ferro-Luzzi A, Helsing E (1995). Mediterranean diet pyramid: a cultural model for healthy eating. Am J Clin Nutr.

[26] Shivappa N, Steck SE, Hurley TG, Hussey JR, Hébert JR (2014). Designing and developing a literature-derived, population-based dietary inflammatory index. Public Health Nutr.

[27] Krebs-Smith SM, Pannucci TE, Subar AF, Kirkpatrick SI, Lerman JL, Tooze JA (2018). Update of the healthy eating index: HEI-2015. J Acad Nutr Dietetics.

[28] Molendijk M, Molero P, Sánchez-Pedreño FO, Van der Does W, Martínez-González MA (2018). Diet quality and depression risk: a systematic review and dose-response meta-analysis of prospective studies. J Affect Disord.

[29] Faghih S, Babajafari S, Mirzaei A, Akhlaghi M (2020). Adherence to the dietary approaches to stop hypertension (DASH) dietary pattern and mental health in iranian university students. Eur J Nutr.

[30] Gianfredi V, Koster A, Odone A, Amerio A, Signorelli C, Schaper NC (2021). Associations of dietary patterns with incident depression: the maastricht study. Nutrients.

[31] Sadeghi O, Keshteli AH, Afshar H, Esmaillzadeh A, Adibi P (2021). Adherence to Mediterranean dietary pattern is inversely associated with depression, anxiety and psychological distress. Nutr Neurosci.

[32] Sánchez-Villegas A, Martínez-González MA, Estruch R, Salas-Salvadó J, Corella D, Covas MI (2013). Mediterranean dietary pattern and depression: the PREDIMED randomized trial. BMC Med.

[33] Phillips CM, Shivappa N, Hébert JR, Perry IJ (2018). Dietary inflammatory index and mental health: a cross-sectional analysis of the relationship with depressive symptoms, anxiety and well-being in adults. Clin Nutr.

[34] Kim ES, Jung BM (2006). A survey of satisfaction and preference for military meal service and food behaviors and food habits of some military personnel. Korean J Community Nutr.

[35] Peduzzi P, Concato J, Kemper E, Holford TR, Feinstein AR (1996). A simulation study of the number of events per variable in logistic regression analysis. J Clin Epidemiol.

[36] Nielsen SJ, Adair L (2007). An alternative to dietary data exclusions. J Am Diet Assoc.

[37] Mirmiran P, Esfahani FH, Mehrabi Y, Hedayati M, Azizi F (2010). Reliability and relative validity of an FFQ for nutrients in the Tehran lipid and glucose study. Public Health Nutr.

[38] Ghaffarpour M, Houshiar-Rad A, Kianfar H. The manual for household measures, cooking yields factors and edible portion of foods. Tehran: Nashre Olume Keshavarzy. 1999;7:213.

[39] Maddahi N, Aghamir SMK, Moddaresi SS, Mirzaei K, Alizadeh S, Yekaninejad MS (2020). The association of dietary approaches to stop hypertension-style diet with urinary risk factors of kidney stones formation in men with nephrolithiasis. Clin Nutr ESPEN.

[40] Trichopoulou A, Lagiou P (1997). Healthy traditional Mediterranean diet: an expression of culture, history, and lifestyle. Nutr Rev.

[41] Shivappa N, Hébert JR, Rietzschel ER, De Buyzere ML, Langlois M, Debruyne E (2015). Associations between dietary inflammatory index and inflammatory markers in the Asklepios Study. Br J Nutr.

[42] Maddahi N, Yarizadeh H, Aghamir SMK, Alizadeh S, Yekaninejad MS, Mirzaei K (2020). The association of dietary inflammatory index with urinary risk factors of kidney stones formation in men with nephrolithiasis. BMC Res Notes.

[43] Sahebi A, Asghari MJ, Salari RS. Validation of depression anxiety and stress scale (DASS-21) for an Iranian population. 2005.

[44] Maddahi N, Setayesh L, Mehranfar S, Alizadeh S, Yekaninejad MS, Mirzaei K (2022). Association of serum levels of vitamin D and vitamin D binding protein with mental health of overweight/obese women: a cross sectional study. Clin Nutr ESPEN.

[45] Maddahi NS, Yarizadeh H, Setayesh L, Nasir Y, Alizadeh S, Mirzaei K (2020). Association between dietary energy density with mental health and sleep quality in women with overweight/obesity. BMC Res Notes.

[46] Moghaddam MB, Aghdam FB, Jafarabadi MA, Allahverdipour H, Nikookheslat SD, Safarpour S (2012). The iranian version of International Physical Activity Questionnaire (IPAQ) in Iran: content and construct validity, factor structure, internal consistency and stability. World Appl Sci J.

[47] Janghorbani M, Amini M, Willett WC, Gouya MM, Delavari A, Alikhani S (2007). First nationwide survey of prevalence of overweight, underweight, and abdominal obesity in iranian adults. Obesity.

[48] Chan R, Chan D, Woo J (2014). A prospective cohort study to examine the association between dietary patterns and depressive symptoms in older chinese people in Hong Kong. PLoS ONE.

[49] Faghih S, Babajafari S, Mirzaei A, Akhlaghi M (2020). Adherence to the dietary approaches to stop hypertension (DASH) dietary pattern and mental health in iranian university students. Eur J Nutr.

[50] Burrows K, Stewart JL, Antonacci C, Kuplicki R, Thompson K, Taylor A (2020). Association of poorer dietary quality and higher dietary inflammation with greater symptom severity in depressed individuals with appetite loss. J Affect Disord.

[51] Haghighatdoost F, Feizi A, Esmaillzadeh A, Feinle-Bisset C, Keshteli AH, Afshar H (2019). Association between the dietary inflammatory index and common mental health disorders profile scores. Clin Nutr.

[52] Bergmans RS, Malecki KM (2017). The association of dietary inflammatory potential with depression and mental well-being among US adults. Prev Med.

[53] Shivappa N, Schoenaker DA, Hebert JR, Mishra GD (2016). Association between inflammatory potential of diet and risk of depression in middle-aged women: the australian longitudinal study on women’s Health. Br J Nutr.

[54] Salari-Moghaddam A, Keshteli AH, Afshar H, Esmaillzadeh A, Adibi P (2021). Empirically derived food-based dietary inflammatory index is associated with increased risk of psychological disorders in women. Nutr Neurosci.

[55] Akbaraly TN, Kerleau C, Wyart M, Chevallier N, Ndiaye L, Shivappa N (2016). Dietary inflammatory index and recurrence of depressive symptoms: results from the Whitehall II study. Clin Psychol Sci.

[56] Wang J, Zhou Y, Chen K, Jing Y, He J, Sun H (2019). Dietary inflammatory index and depression: a meta-analysis. Public Health Nutr.

[57] Shafiei F, Salari-Moghaddam A, Larijani B, Esmaillzadeh A (2019). Adherence to the Mediterranean diet and risk of depression: a systematic review and updated meta-analysis of observational studies. Nutr Rev.

[58] Lai JS, Oldmeadow C, Hure AJ, McEvoy M, Byles J, Attia J (2016). Longitudinal diet quality is not associated with depressive symptoms in a cohort of middle-aged australian women. Br J Nutr.

[59] Rienks J, Dobson A, Mishra G (2013). Mediterranean dietary pattern and prevalence and incidence of depressive symptoms in mid-aged women: results from a large community-based prospective study. Eur J Clin Nutr.

[60] Gibson-Smith D, Bot M, Brouwer IA, Visser M, Penninx BW (2018). Diet quality in persons with and without depressive and anxiety disorders. J Psychiatr Res.

[61] Beydoun MA, Kuczmarski MTF, Mason MA, Ling SM, Evans MK, Zonderman AB (2009). Role of depressive symptoms in explaining socioeconomic status disparities in dietary quality and central adiposity among US adults: a structural equation modeling approach. Am J Clin Nutr.

[62] Rahe C, Baune BT, Unrath M, Arolt V, Wellmann J, Wersching H (2015). Associations between depression subtypes, depression severity and diet quality: cross-sectional findings from the BiDirect Study. BMC Psychiatry.

[63] Akbaraly TN, Sabia S, Shipley MJ, Batty GD, Kivimaki M (2013). Adherence to healthy dietary guidelines and future depressive symptoms: evidence for sex differentials in the Whitehall II study. Am J Clin Nutr.

[64] Recchia D, Baghdadli A, Lassale C, Brunner E, Verdier J-M, Kivimäki M (2020). Associations between long-term adherence to healthy diet and recurrent depressive symptoms in Whitehall II study. Eur J Nutr.

[65] Gougeon L. Nutritional predictors of depression in a cohort of community-dwelling elderly Canadians. NuAge cohort; 2014.

[66] Lai JS (2015). A systematic review and meta-analysis of dietary patterns and depression in community-dwelling adults. Am J Clin Nutr.

[67] Elhwuegi AS (2004). Central monoamines and their role in major depression. Prog Neuropsychopharmacol Biol Psychiatry.

