# Supplementary material for: The association between a priori dietary patterns and psychological disorders in military personnel
Source: BMC Psychiatry. 2023 Mar 28;23:203. doi: 10.1186/s12888-023-04650-x (PMC10053979; doi:10.1186/s12888-023-04650-x)
Supplement: Supplementary file 1 — Supplementary Material 1 Supplemental Table 1. Food intakes (macronutrients and micronutrients) of participants in first and last quartiles of dietary inflammatory index (DII) [file 12888_2023_4650_MOESM1_ESM.docx]

Supplemental Table 1. Food intakes (macronutrients and micronutrients) of participants in
first and last quartiles of dietary inflammatory index (DII)

|  |  | Quartiles of DII | | |
| --- | --- | --- | --- | --- |
|  | Total  (n=400) | Q1  (n= 100) | Q4  (n= 100) | P value* |
| Energy (kcal) | 2169.85±806.19 | 1349.30±318.83 | 3044.56 ±570.83 | ≤0.001 |
| Protein (g) | 96.08±123.30 | 46.64±14.45 | 182.75±221.11 | ≤0.001 |
| Carbohydrate (g) | 388.97±394.44 | 182.05±50.66 | 744.49±645.58 | ≤0.001 |
| Total fat (g) | 86.84±56.14 | 47.02±17.82 | 144.37±72.48 | ≤0.001 |
| Fiber (g) | 37.39±32.67 | 72.85±48.54 | 17.41±4.68 | ≤0.001 |
| Monounsaturated fatty acids (g) | 31.89±41.65 | 59.65±74.65 | 15.67±6.09 | ≤0.001 |
| Polyunsaturated fatty acids (g) | 17.62±21.66 | 32.44±37.99 | 8.71±4.23 | ≤0.001 |
| Omega-3 fatty acids (g) | 1.09±1.43 | 2.05±2.57 | 0.56±0.27 | ≤0.001 |
| Omega-6 fatty acids (g) | 15.32±18.99 | 28.02±33.19 | 7.57±3.98 | ≤0.001 |
| Saturated fatty acids (g) | 28.40±20.41 | 15.35±7.23 | 48.35±28.08 | ≤0.001 |
| Cholesterol (mg) | 614.20±1465.78 | 324.72±195.51 | 1177.80±2802.54 | ≤0.001 |
| Iron (mg) | 37.65±52.70 | 17.52±7.52 | 72.85±94.96 | ≤0.001 |
| Zinc (mg) | 21.62±22.36 | 41.42±36.80 | 9.82±2.74 | ≤0.001 |
| Vitamin C (mg) | 356.54±417.42 | 788.87±645.59 | 134.11±58.40 | ≤0.001 |
| Cobalamin (μg) | 12.72±76.37 | 30.59±151.51 | 4.79±2.42 | ≤0.001 |
| Vitamin A (RAE) | 1800.16±7515.52 | 4474.13±14725.25 | 597.52±244.47 | ≤0.001 |
| Thiamin (mg) | 1.61±1.57 | 2.90±2.63 | 0.86±0.27 | ≤0.001 |
| Riboflavin (mg) | 2.63±5.63 | 5.24±10.81 | 1.18±0.42 | ≤0.001 |
| Pyridoxine (mg) | 2.28±2.57 | 4.50±4.36 | 1.02±0.28 | ≤0.001 |
| Folate (μg) | 537.41±497.38 | 980.30±817.83 | 281.61±82.57 | ≤0.001 |
| Niacin (mg) | 23.90±38.67 | 45.76±55.61 | 11.76±3.41 | ≤0.001 |
| Magnesium (mg) | 383.03±166.94 | 398.89±167.60 | 382.01±168.32 | 0.65 |
| Selenium (mg) | 56.01±12.63 | 65.41±21.58 | 49.92±2.27 | ≤0.001 |
| Vitamin E (mg) | 12.26±11.92 | 22.66±19.36 | 5.75±2.71 | ≤0.001 |
| Vitamin D (μg) | 2.95±3.12 | 5.05±4.82 | 1.46±1.22 | ≤0.001 |
| Vitamin K (μg) | 458.67±612.31 | 946.46±1040.31 | 191.03±113.75 | ≤0.001 |

* P values are extracted from ANOVA analysis.
